# SeqBreed: a python tool to evaluate genomic prediction in complex scenarios

**DOI:** 10.1186/s12711-020-0530-2

**Published:** 2020-02-10

**Authors:** Miguel Pérez-Enciso, Lino C. Ramírez-Ayala, Laura M. Zingaretti

**Affiliations:** 1 Centre for Research in Agricultural Genomics (CRAG), CSIC-IRTA-UAB-UB, 08193 Bellaterra, Barcelona Spain; 2 ICREA, Passeig de Lluís Companys 23, 08010 Barcelona, Spain; 3 Universidad Nacional de Villa María, IAPBCyA-IAPCH Villa María, Córdoba, Argentina

## Abstract

**Background:**

Genomic prediction (GP) is a method whereby DNA polymorphism information is used to predict breeding values for complex traits. Although GP can significantly enhance predictive accuracy, it can be expensive and difficult to implement. To help design optimum breeding programs and experiments, including genome-wide association studies and genomic selection experiments, we have developed SeqBreed, a generic and flexible forward simulator programmed in python3.

**Results:**

SeqBreed accommodates sex and mitochondrion chromosomes as well as autopolyploidy. It can simulate any number of complex phenotypes that are determined by any number of causal loci. SeqBreed implements several GP methods, including genomic best linear unbiased prediction (GBLUP), single-step GBLUP, pedigree-based BLUP, and mass selection. We illustrate its functionality with Drosophila genome reference panel (DGRP) sequence data and with tetraploid potato genotype data.

**Conclusions:**

SeqBreed is a flexible and easy to use tool that can be used to optimize GP or genome-wide association studies. It incorporates some of the most popular GP methods and includes several visualization tools. Code is open and can be freely modified. Software, documentation, and examples are available at https://github.com/miguelperezenciso/SeqBreed.

## Background

Genomic prediction (GP) is a method whereby DNA polymorphism information is used to predict the breeding value of individuals for complex traits. The availability of high-throughput single nucleotide polymorphism (SNP) genotyping in a cost-effective manner has led GP to become a standard tool in the analysis and improvement of complex traits [1]. GP has revolutionized breeding programs in plants and animals and, today, GP methods are also widely used in human genetics or ecology. Nevertheless, GP is more expensive than traditional pedigree-based breeding. GP can be difficult to implement in practical scenarios, due in part to the difficulty of optimizing genotyping strategies and to uncertainty about the genetic basis of complex traits. Thus, it is highly advisable to evaluate its potential advantages and expected performance in advance. GP accuracy depends on a large number of factors. Several of these can be controlled by the practitioner, to some extent, such as the number of SNPs, number of individuals, selection intensity, and the evaluation method. Other factors cannot be modified, such as linkage disequilibrium and are even unknown (genetic architecture). Although several approximations of the accuracy of GP have been developed, e.g. [2, 3], it remains difficult to analytically assess the influence of these factors in practical scenarios across generations. For this purpose, stochastic computer simulation is the most reliable option. Although critical factors such as the detailed genetic architecture of complex traits are unknown, the main genetic parameters are reasonably well known for most complex traits, such as heritability and the distribution of genetic effects, which can be approximated by a gamma distribution [4–6]. Thus, a simulation study can be performed to evaluate the effect of the number of causal loci quantitative trait nucleotides (QTN) and of their location to assess the robustness of predictions.

Here, we present a versatile python3 forward simulation tool, SeqBreed, to evaluate GP performance in generic scenarios and with any genetic architecture (i.e., number of QTN, their effects and location, and the number of traits). The purpose of SeqBreed is to generate phenotype and genotype data of individuals under different (genomic) selection strategies. SeqBreed is inspired by a previous pSBVB fortran software program [7], but the code has been rewritten in python3 and many new options have been added. Python can be much slower than compiled languages, but is much easier and friendlier to use, allowing direct interaction with the user to, e.g., make plots or control selection and breeding decisions. In addition, many libraries in python, such as ‘numpy’ (https://numpy.org/) or ‘pandas’ (https://pandas.pydata.org/), are wrappers on compiled languages, such that careful programming significantly alleviates the limited speed of native python. Thus, SeqBreed is much more versatile than pSBVB and incorporates many new options, such as genome-wide association studies (GWAS) and principal component analysis (PCA). Most importantly, it allows automatic implementation of standard genomic selection procedures. Usage details and the main features of SeqBreed are described in the following and in the accompanying GitHub site https://github.com/miguelperezenciso/SeqBreed.

## Implementation

### Outline

Broadly, SeqBreed takes genotype/sequence data from a founder population and simulates phenotypes according to a predetermined genetic architecture. Offspring genomes and phenotypes can be simulated under selection or random drift. By default, selection is simulated across a predetermined number of generations and selection intensities. SeqBreed offers extensive flexibility to the user. For example, accuracy of GP with several SNP arrays can be simultaneously compared using the same data; offspring of specific pairs of parents can be generated; and dihaploid offspring can be simulated. SeqBreed can be run using scripts or interactively, where the user can, say, obtain plots for each generation or generate genotype data of a given set of individuals. Examples of the program’s usage are in the GitHub’s jupyter notebook https://github.com/miguelperezenciso/SeqBreed/blob/master/SeqBreed_tutorial.ipynb and in the python script https://github.com/miguelperezenciso/SeqBreed/blob/master/main.py.

**Fig. 1:**
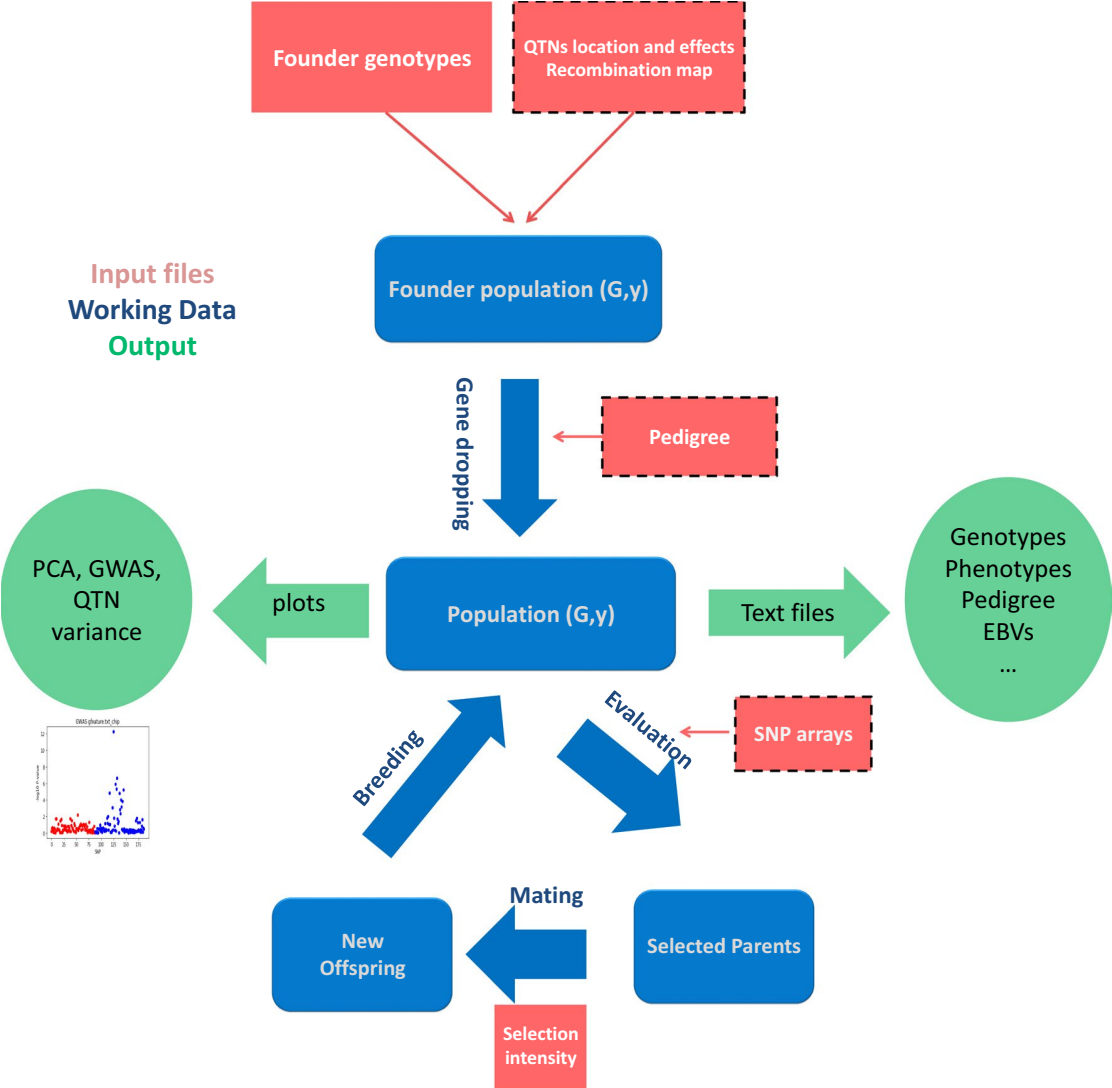
Outline of the SeqBreed pipeline. Inputs are shown in red squares, dashed border rectangles represent optional input, internal data are in blue rounded squares, main operations are indicated in blue, and outputs are in green circles; G and y refer to genotypes and phenotypes, respectively. The program starts with an optional gene dropping step following an input pedigree. No selection is performed at this stage. The bottom loop represents selection, where new offspring are generated based on the genotypes of selected parents. A list of SNPs in the genotyping array must be determined when using GBLUP and BLUP. A new cycle starts when these new offspring are added to the existing population. Plots can be performed at several stages

SeqBreed is programmed in python3 using an object-oriented paradigm. The generic SeqBreed flowchart is visualized in Fig. 1. As input, SeqBreed minimally requires a genotype file from the founder base population in vcf [8] or plink-like format [9]. A typical SeqBreed run consists of the following steps:

1. Upload founder sequence genotypes in vcf or plink format. The program automatically determines ploidy and the number of chromosomes and SNPs.
2. Specify genome characteristics. Sex-linked SNPs and/or recombination rates can be specified.
3. Specify desired heritabilities and causal SNPs (QTN) and their effects for every trait. Environmental variances are inferred given founder genomes, QTN effects and heritabilities.
4. Offspring genomes and phenotypes are simulated by gene-dropping along a predetermined pedigree or by implementing selection.

PCA plots or GWAS options are also implemented. The main python classes are:

- Population: This class contains the main attributes for running selection experiments and is a container for Individual objects. It includes methods to add new individuals generated by mating two parents or randomly shuffling founder genomes in order to increase the number of base population animals (see [10]). It also prints basic population data and summary plots.
- Individual: It allows generation, manipulation, and printing of the genotypes and phenotypes of individuals. Internally, an individual’s genome is represented by contiguous non recombining blocks rather than by the list of all SNP alleles, which allows dramatic savings in memory and increases in efficiency (see Figure 1 in Pérez-Enciso et al. [10]).
- Genome: All genome characteristics are stored and can be accessed by methods in this class. It specifies ploidy, number and class of chromosomes, and recombination rates or SNP positions.
- GFounder: SeqBreed requires as minimum input the genotypes of the so-called ‘founder population’, which comprises the parents of the rest of the individuals to be generated. This class stores these genotypes and automatically retrieves main genome features such as SNP positions, number of chromosomes, etc. Initial genotypes can be filtered by minimum allele frequency (MAF).
- QTN: This class determines the genetic architecture for each trait simulated. It has methods to determine the environmental variance given a desired heritability, and to plot variance components for QTN. In its current version, SeqBreed allows for dominance and additive actions, but not epistasis.
- Chip: This class is basically a container for the list of SNPs that are included in a genotyping array. It allows easy comparison of different genotyping strategies in genomic selection.

### Specifying genome features and genetic architecture

By default, SeqBreed assumes that all loci are autosomal and a recombination rate of 1 cM = 1 Mb throughout the genome. It includes options to specify sex or mitochondrial chromosomes, and local and sex specific recombination maps. A pseudo-autosomal region (PAR) is not accommodated for sex chromosomes, i.e., the whole Y chromosome is assumed to be non-recombining. A mitochondrial chromosome is a non-recombining chromosome that is transmitted maternally. SeqBreed allows for autopolyploidy of any level, which is automatically detected from vcf files. Accurate modeling of meiosis in polyploids is notoriously difficult [11, 12] and SeqBreed implements a simplified algorithm:

1. For each chromosome id, homologs are randomly paired.
2. Within each pair of homologs, cross-over events are generated as for diploids, i.e., no interaction between homologous chromosomes is modeled and the number of cross-over events is simulated following a Poisson distribution with a rate equal to chromosome length in Morgans.
3. Sex chromosomes are modeled with a maximum ploidy of 2.

Therefore, our algorithm does not fully model the interaction of preferential pairing of homologous chromosomes and double reduction arising from multivalent formation [13]. For the purposes of this software (i.e. comparison of GP strategies over a limited number of generations), it is unlikely that this approximation has a dramatic effect.

SeqBreed allows the simulation of any number of phenotypic traits, regardless of ploidy. For each trait, broad-sense heritability must be specified. There are three options to specify the number of QTN and their effects (https://github.com/miguelperezenciso/SeqBreed#3-specifying-genetic-architecture): (i) a random number of QTN positions are sampled genome-wide and additive effects are sampled from a gamma distribution $\Gamma$ (shape = 0.2 and scale = 5), as suggested by Caballero et al. [5]; (ii) the positions of the QTN are specified in a file and additive effects are sampled from a gamma distribution; and (iii) QTN positions and additive and dominant effects for each trait are specified in an external file. By default, QTN are not removed from the sequence data to perform genomic evaluation. To remove QTN from evaluation, a SNP chip can be defined that excludes the QTN. Options (i) and (ii) can only be used with one trait and without dominance. SeqBreed adjusts the environmental variance $Var\left( e \right)$ to retrieve the desired broad-sense heritabilities (${\text{H}}^{2}$) from $Var\left( e \right) = Var\left( g \right) \times \left( {1 - {\text{H}}^{2} } \right)/{\text{H}}^{2}$, where $Var\left( g \right)$ is the variance of the genotypic values of individuals in the founder population. The genotypic value for individual $i$ is defined as:

$$g_{i} = \mathop \sum \limits_{j = 1}^{nQTN} \gamma_{ij } a_{j} + \mathop \sum \limits_{j = 1}^{nQTN} \delta_{ij } d_{j} ,$$

where $nQTN$ is the number of QTN, $a_{j}$ is the additive effect of the $j$-th QTN, that is, half the expected difference between homozygous genotypes, with $\gamma_{ij }$ taking the values − 1, 0 and 1 for homozygous, heterozygous, and alternative homozygous genotypes, respectively, $d_{j}$ is the dominance effect of the $j$-th QTN, with $\delta_{ij }$ taking the value 1 if the genotype is heterozygous and 0 otherwise. In the case of polyploids:

$$g_{i} = \mathop \sum \limits_{j = 1}^{nQTN} \eta_{ij } a_{j} + \mathop \sum \limits_{j = 1}^{nQTN} \varphi_{ij } d_{j} ,$$

where $\eta_{ij }$ is the number of copies of the alternative allele (coded as 1) minus half the ploidy for the $j$-th QTN and the $i$-th individual, and $a_{j}$ is therefore the expected change in phenotype per copy of allele ‘1’ at the $j$-th QTN. In polyploids, technically as many dominance coefficients as ploidy levels (h) minus two can be defined, which is not practical. As in pSBVB [7], we define $\varphi_{ij }$ as the minimum number of copies of allele ‘1’ such that the expected phenotype is $d$ (see Figure 1 in Zingaretti et al. [7]). SeqBreed uses $\varphi_{ij } = 1$, that is, all heterozygous individuals have the same genotype value as the complete homozygous ‘1’. SeqBreed computes genotypic values for each individual and simulate phenotypes from $y_{i} = \mu + g_{i} + e_{i}$, where $\mu$ is a constant and $e$ is a normal deviate $e\sim N\left( {0,Var\left( e \right)} \right)$.

For multiple traits, the user needs to specify additive and dominant QTN effects separately for each trait. This is done via an external text file, where additive and dominant QTN effects are specified for each trait (option 3 in https://github.com/miguelperezenciso/SeqBreed#3-specifying-genetic-architecture). There is no specific assumption on genetic correlations between traits. To simulate no pleiotropy, QTN for each trait have zero effects for all other traits. Note that this does not prevent a non-zero genetic correlation arising from linkage disequilibrium. The program does not automatically adjust desired genetic correlations and, thus, different QTN values may need to be tested to fit desired correlations. Environmental correlations are always zero.

It is typically difficult to find real sequence data to generate a reasonably sized founder population. To accommodate this, SeqBreed can generate ‘dummy’ founder individuals by randomly combining recombinant haplotypes. This can be done in two ways, either by generating a random pedigree and simulating a new founder individual by gene-dropping along this pedigree, or by directly simulating a number of recombining breakpoints and assigning random founder genotypes to each block between recombination breakpoints (https://github.com/miguelperezenciso/SeqBreed/blob/master/README.md#breeding-population).

### Gene dropping and selection implementation

Seqbreed can be run along a predetermined pedigree or using a combination of options (several examples are provided in the GitHub site). It is also possible to generate new individuals interactively, including dihaploids. To speed up computations and avoid unnecessary memory usage, only recombination breaks and ancestor haplotype ids are stored for each individual (see Figure 1 in [14]).

SeqBreed allows computing estimated breeding values using several GP methods. It also allows several lists of SNPs (SNP chips) to be defined, such that GP performance can be easily compared across chips. From a methodological point of view, most GP implementations are based on penalized linear methods (e.g., de los Campos et al. [15]). SeqBreed includes some of the most popular GP options, including pedigree BLUP [16], GBLUP [17], and single-step GBLUP [18]. Only single trait GP algorithms are implemented so far. Mass selection is also implemented. For GBLUP and single-step GBLUP, the genomic relationship matrix ${\mathbf{G}}$ is obtained using VanRaden [17] as:

$${\mathbf{G}} = \frac{{{\mathbf{XX}} '}}{{2\mathop \sum \nolimits_{j = 1}^{nSNP} p_{j} \left( {1 - p_{j} } \right)}},$$

where ${\mathbf{X}}$ is a $N \times n{\text{SNP}}$ matrix containing genotypes (coded 0,1,2 deviated from the mean) for SNPs on the chip, $p_{j}$ is the allele frequency of the $j$-th SNP, $N$ is the number of individuals, and $n{\text{SNP}}$ is the number of SNPs. To avoid potential singularity problems, diagonal elements of ${\mathbf{G}}$ are multiplied by 1.05. SeqBreed requires that heritabilities to be used in BLUP or GBLUP are provided (i.e., they are not estimated). The program allows the incorporation of other custom GP methods based on a user python function or by exporting SNP data and phenotypes from SeqBreed, running a genetic evaluation, externally and then importing the resulting estimated breeding values.

Selection can be automatically configured and run, as documented in the GitHub examples (https://github.com/miguelperezenciso/SeqBreed). Running a selection scheme requires specifying the number of generations, the numbers of females and males to be selected, and the number of offspring per female. SeqBreed splits the selection process in three steps, which allows a fine control over the breeding program. First, breeding values are predicted using the chosen evaluation method and marker information. By default, the data from all individuals across the current and previous generations are used, but this can be changed by specifying the subset of individuals to be used. Second, a function is used to generate offspring from selected parents. This function requires specifying the candidates for selection (allowing for continuous or discrete generations), selection intensity, family size, and either assortative or random mating between selected parents. Hierarchical mating between females and males is employed by default (https://github.com/miguelperezenciso/SeqBreed#7-implementing-selection). Assortative and random mating schemes are implemented; more sophisticated mating schemes, such as those based on optimal contributions [19, 20], have to be specified manually by modifying the function ‘ReturnNewPed’ in the selection module (https://github.com/miguelperezenciso/SeqBreed/blob/master/src/selection.py).

### Visualization

**Fig. 2:**
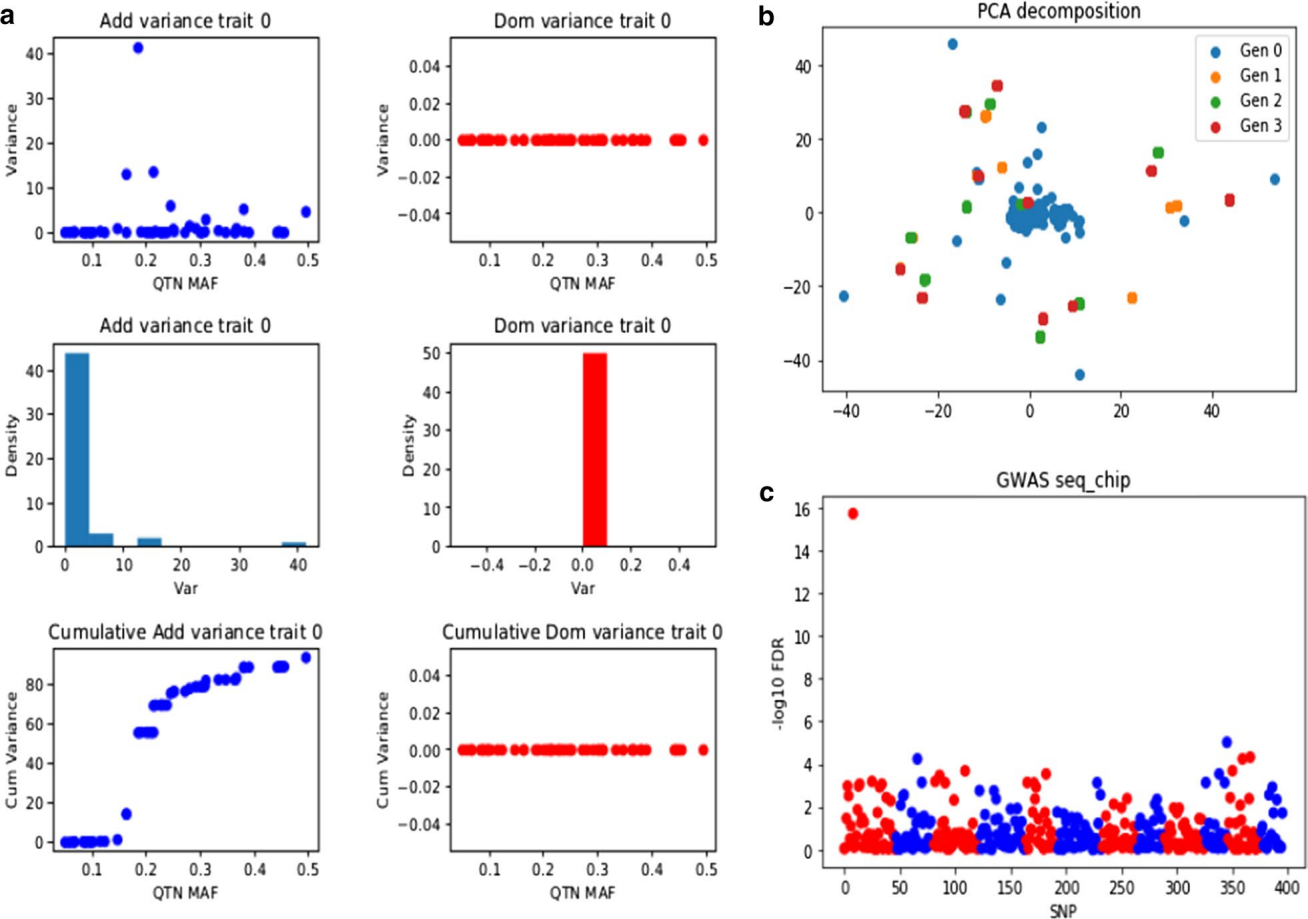
Example plots produced by SeqBreed. **a** Contribution of each QTN to total variance. Top, individual QTN variances as a function of minimum allele frequency (MAF); middle, histogram of QTN variances; bottom, cumulative variance when QTN are sorted by MAF. In blue, additive variances; in red, dominance variances. The figure shows a fully additive phenotype, thus dominance variances are zero. **b** Principal component analysis plot; individuals of different generations are in different colors. **c** Genome-wide association study showing false discovery rate values (− log10 scale). SNPs on different chromosomes are represented in alternate colors

A novel feature of SeqBreed, as compared to our previous software pSBVB, is the capability of graphical outputs. Figure 2 illustrates some of the plots that can be performed automatically. Figure 2a shows the results of the QTN.plot() function, which plots the individual QTN variance as a function of MAF, the histogram of QTN variances, and the cumulative variance when QTN are sorted by MAF. This is performed for each phenotype and for both additive and dominance variances, based on allele substitution effects $\alpha = a + d\left( {1 - 2p} \right)$, where $p$ is the minimum allele frequency, and assuming complete equilibrium. In addition, PCA plots using all sequence or custom defined SNP sets (Fig. 2b) are available, as well as GWAS plots showing p-values or false discovery rate (FDR) values (Fig. 2c). Genotype and phenotype data can also be exported in text files.

### Usage and examples

The basic functioning of SeqBreed is illustrated by the main.py script that is available at https://github.com/miguelperezenciso/SeqBreed/blob/master/main.py. This script, or its equivalent jupyter notebook (SeqBreed_tutorial.ipynb), shows the basic commands to run SeqBreed and import the required modules. First, SeqBreed modules are imported as:
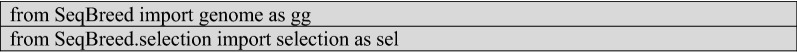


Founder population genotypes are uploaded from the vcf file, using the command:
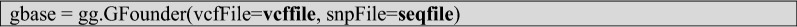


which generates a GFounder object that contains founder genotypes, vcffile is the file containing genotypes and seqfile is generated by the program and contains information about SNP positions, which are used in the next step.

Next, the main genome features are specified. The following command creates a Genome object that assumes that the ‘X’ chromosome is the sex X chromosome, while SNPs on the chromosome named ‘MT’ are mitochondrial.
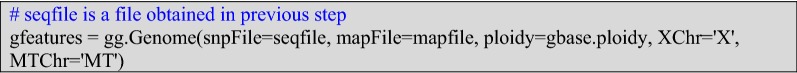


Genetic architecture can be specified in different ways. The simplest is to generate nqtn QTN randomly distributed along the genome with effects sampled from a gamma distribution, where h2 is the desired heritability.
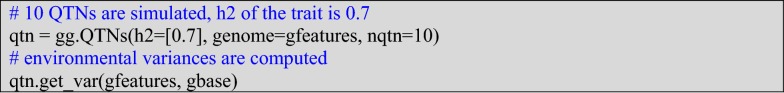


Selection is implemented in cycles, the number of generations, the numbers of males and females selected, and family size must be specified. The following is an example with GBLUP selection, random mating, and continuous generations.
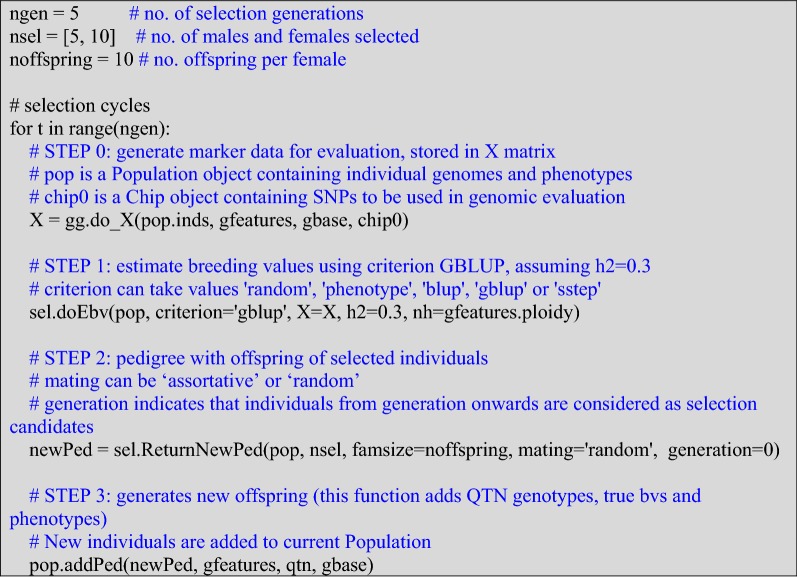


We illustrate the software with sequence data from the Drosophila genome reference panel (DGRP, [21]), parsed and filtered as explained in [22], and genotype data from tetraploid potato [23], parsed as described in [7]. Data and scripts are in https://github.com/miguelperezenciso/SeqBreed/tree/master/DGRP and in https://github.com/miguelperezenciso/SeqBreed/tree/master/POTATO for the Drosophila and potato examples, respectively. The DGRP scripts illustrate the specific recombination map of Drosophila, where males do not recombine, as shown in the ‘dgrp.map’ file. The example provided in GitHub consists of a small experiment to compare genomic and mass selection. Plots in the jupyter notebook are implemented to track phenotypic changes by generation. The potato scripts illustrate how to generate an F2 cross between extreme lines and to perform a GWAS experiment in polyploids. GWAS results using PCA corrected phenotypes are also shown.

## Conclusions and future developments

Several other programs have been developed for similar purposes as SeqBreed, including our own pSBVB [7], AlphaSim [24] and its successor AlphaSimR (https://alphagenes.roslin.ed.ac.uk/wp/software-2/alphasimr/), PedigreeSim [13], simuPOP [25], and QMSim [26]. However, SeqBreed offers a unique combination of features for simulation of GP of complex traits, including built-in implementation of several GP methods, the possibility of simulating polyploid genomes, and several options to specify QTN and SNP arrays. It also allows new individuals to be generated interactively and provides graphical plots of results. It is easy to use, easy to install, and software options are illustrated with several examples in the GitHub site. Given the interactive nature of python and its graphical features, SeqBreed is especially suited for educational purposes. However, for large-scale simulations SeqBreed will not be as efficient as some Fortran counterparts such as AlphaSim or pSBVB.

Note that SeqBreed was designed to evaluate the performance of GP or GWAS over a short time horizon, i.e., new mutations are not generated. SeqBreed is not designed to investigate the long-term effects of demography or selection on DNA variability because new mutations are not generated. For these purposes, Slim [27] or similar tools are more appropriate. To investigate realistic scenarios, the recommended input for SeqBreed is real sequence data.

Plans for further development of SeqBreed include additional features to generalize available genetic architectures (e.g., imprinting, epistasis), integration with machine-learning tools (scikit, keras) for genetic evaluation, development of an educational tool with an html-based interface, and improving output and plotting features.

## Data Availability

https://github.com/miguelperezenciso/SeqBreed Availability and requirements: Project name: SeqBreed. Project home page: https://github.com/miguelperezenciso/SeqBreed. Operating systems: Tested in linux and mac. It should also run in windows python. Programming language: Python. License: GNU GPLv3. Any restrictions to use by non-academics: None.
